# Proteomic analysis of *Staphylococcus aureus* biofilm cells grown under physiologically relevant fluid shear stress conditions

**DOI:** 10.1186/1477-5956-12-21

**Published:** 2014-04-30

**Authors:** Nazrul Islam, Yonghyun Kim, Julia M Ross, Mark R Marten

**Affiliations:** 1 Department of Chemical, Biochemical and Environmental Engineering, University of Maryland Baltimore County (UMBC), Baltimore, MD 21250, USA; 2 Department of Chemical and Biological Engineering, The University of Alabama, Tuscaloosa, AL 35487, USA; 3 Current address: Department of Plant Sciences, University of Maryland, College Park, MD 20742, USA

**Keywords:** Biofilm, *Staphylococcus aureus*, Flow chamber, Shear stress, Proteomics

## Abstract

**Background:**

The biofilm forming bacterium *Staphylococcus aureus* is responsible for maladies ranging from severe skin infection to major diseases such as bacteremia, endocarditis and osteomyelitis. A flow displacement system was used to grow *S. aureus* biofilms in four physiologically relevant fluid shear rates (50, 100, 500 and 1000 s^-1^) to identify proteins that are associated with biofilm.

**Results:**

Global protein expressions from the membrane and cytosolic fractions of *S. aureus* biofilm cells grown under the above shear rate conditions are reported. Sixteen proteins in the membrane-enriched fraction and eight proteins in the cytosolic fraction showed significantly altered expression (*p* < 0.05) under increasing fluid shear. These 24 proteins were identified using nano-LC-ESI-MS/MS. They were found to be associated with various metabolic functions such as glycolysis / TCA pathways, protein synthesis and stress tolerance. Increased fluid shear stress did not influence the expression of two important surface binding proteins: fibronectin-binding and collagen-binding proteins.

**Conclusions:**

The reported data suggest that while the general metabolic function of the sessile bacteria is minimal under high fluid shear stress conditions, they seem to retain the binding capacity to initiate new infections.

## Background

A biofilm is an aggregate of bacterial microcolonies adherent to each other and to biotic or abiotic surfaces, embedded in an extracellular matrix produced by the sessile bacteria composing the microcolonies. Biofilm has been found in 65-80% of the bacterial infections, and is considered refractory to host defenses in antibiotic therapy [1]. Among the biofilm forming bacteria, *Staphylococcus aureus* is responsible for severe skin infections to such major diseases as bacteremia, endocarditis and osteomyelitis. Under suitable conditions, *S. aureus* causes serious complications in devices like implants and catheters by producing biofilms on them [2,3]. Treatment of such infections becomes even more challenging given that several *S. aureus* strains show resistance to multiple antibiotics (e.g., methicilin and vancomycin). Donlan and Costerton [4] postulated three mechanisms responsible for antibiotic resistance: (i) delayed penetration of the antimicrobial agent through the biofilm matrix, (ii) altered growth rate of biofilm organisms, and (iii) other physiological changes due to the biofilm mode of growth. Growth rate and physiological changes are considered the major causes of resistance to antibiotics. Characterizing bacterial biofilm architecture and stability in their favorable growth conditions would help elucidate how these bacteria survive during their processes of biofilm formation.

In general, increased antimicrobial tolerance is related to the biofilm physiology and architecture [5]. Architecture depends on (i) the development of an extracellular matrix composed of proteins, DNAs, minerals and/or polysaccharides secreted by the sessile cells and (ii) the shear environment in which matrix development occurs [4]. The stability of the structure lies in its viscoelastic properties and its related ability to withstand mechanical cleansing forces (e.g., fluid shear stress) [6]. A number of previous studies investigated the mechanism of adhesion and erosion of *S. aureus* biofilm cells under physiologically relevant fluid shear conditions [7-13]. The latter study demonstrated that platelets (thrombocytes) adhere to the collagen (fibrous structured proteins in blood vessels) of the vascular sub-endothelium in distinct patterns. Shear rates of ≤800 s^-1^ were demonstrated to allow better adhesion than the higher shear rates.

In addition to the above, several studies focused on the protein profiles of *S. aureus* biofilm cells under different growth conditions [14-16]. Higher levels of surface proteins, especially the fibrinogen-binding proteins, were observed in biofilm cells when compared to planktonic cells [17]. Similarly, several other studies investigated protein profile of *S. aureus* biofilm cells under different growth conditions most of which employed either shake flasks or nutrient-rich agar plates to produce biofilm [14-16]. Nonetheless, use of shake flasks or agar plates does not seem to provide appropriate environment for the physiologically relevant biofilm studies for several reasons [17,18]. First, it exposes the bacteria to nutritionally rich media. Second, biofilm grown in shake flasks encounters high osmolarity, oxygen limitation and a relatively high cell density that together influence gene expression in *S. aureus*[19-21]. Third, shake flasks do not mimic the *in-vivo* environment where *Staphylococci* grow as biofilm under fluid shear.

Investigations discussed thus far provided significant information to the molecular mechanisms of biofilm formation, erosion and dissemination. However, little is known how the overall protein profiles in *S. aureus* biofilm cells are influenced under different fluid shear rates. It is hypothesized that fluid shear stress influences gene regulatory networks controlling protein expression and thereby contributing to bacterial adhesion/survival during infection. In this manuscript, a proteomic approach to study the expression of membrane and cytosolic protein fractions in biofilm cells grown under physiologically relevant fluid shears is reported. The protein expression profiles of biofilm cells under different shear conditions provide novel insights into the cellular processes related to bacterial adhesions/survival during infection *in vivo*.

## Results and discussion

### 2-DE profile, protein identification and classification

**Figure 1:**
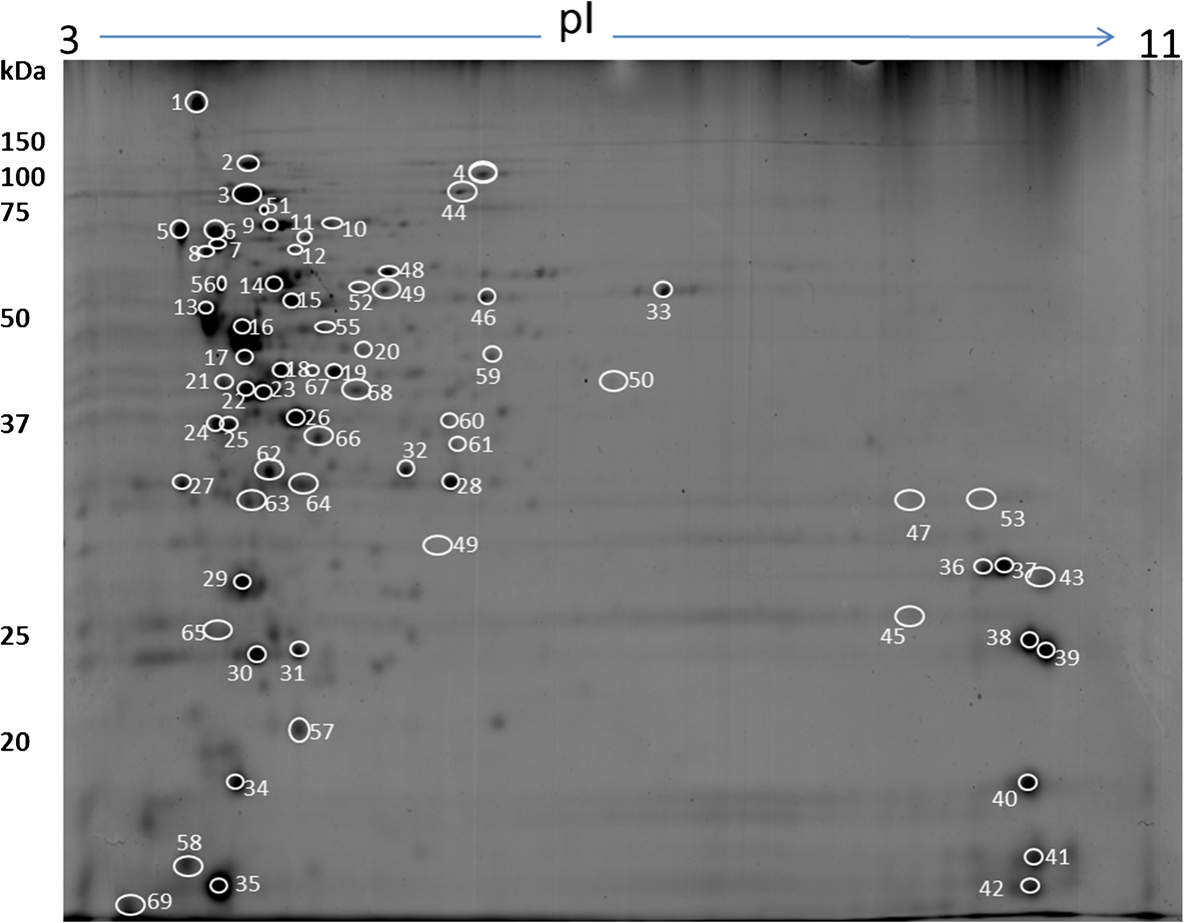
**A 2-DE gel image of *Staphylococcus aureus*.** Membrane protein fraction of bacteria grown in shake flasks for 2 hrs was extracted by digesting with lysostaphin in presence of raffinose. Numbered proteins were identified by nano-LC ESI MS and shown in Table 1.

For the comparison of protein profiles under different shear rates, a two dimensional electrophoresis (2-DE) profile was generated using membrane protein fractions from cells grown in shake flasks for two hours. It is known that most of the proteins associated with bacterial adhesion to the substratum are located in the membrane fraction. Of the 200 ± 5 protein spots resolved in four biological replicate gels, 64 were confidently identified using nano-LC-MS/MS (Figure 1 and Table 1). The remaining 136 protein spots could not be identified due to their low abundance and were below the limit of identification. As shown in Table 1, most of the proteins were identified with high XC score and sequence coverage. Using PSORT [22] and TMpred [23], the subcellular location of 16 proteins were identified as membrane and 20 proteins as transmembrane. Twenty-six proteins were also predicted to be of cytoplasmic origin, which may have originated from lysed cells. The exact reason for the contamination of proteins from cytoplasmic origin is not clear. One possible reason is the inherent chemistry of lysostaphin, the enzyme used in digesting the membrane fraction. A recent study reported that the treatment of bacterial cells with lysostaphin decreases cell wall stiffness by digesting peptidoglycan and could eventually lead to the formation of osmotically fragile cells [24]. A detailed explanation of how lysostaphin causes cell autolysis during digestion is available elsewhere [25]. Alternatively, there is immunological and biochemical evidence that some of the cytoplasmic proteins may be recycled between the cytosol and membrane compartments to perform their role as a cellular scavenger of degradable proteins and carrier of newly synthesized proteins [26]. Similar to this study, contaminations of cytosolic proteins to membrane fractions in bacterial protein extraction were reported elsewhere [25,27-29]. Regardless of the origins, the presence of these cytoplasmic proteins does not diminish the significance of the membrane proteins that were identified in this study. In fact, there are reports that suggest that some of the *S. aureus* cytoplasmic proteins may have a “moonlighting function” and play roles in virulence [30,31].

**Table 1:** Proteins shown in the reference map were identified with a linear ion trap nano-LC-ESI-MS/MS

**Spot ID**	**Protein name**	**Functional category**	**XC-score**	**pI/Mr**	**Cov (%)**	**Accession number**	**Location**
1	Fibronectin-binding protein	Virulence mechanism	150	4.4/114	18	gi:15925493	Membrane
2	Aconitate hydratase	Carbohydrate metabolism	396	4.7/98	51	gi:15924340	transmembrane
3	Elongation factor G	Transcription and replication	548	4.6/77	49	gi:15923537	transmembrane
4	Collagen Binding Protein (Cna)	Virulence mechanism	90	5.8/133	11	gi:21284341	Membrane
5	Trigger factor	Virulence mechanism	140	4.2/49	36	gi:15924665	Cytosolic
6	DnaK protein	Stress protein	348	4.5/66	67	gi:15924570	Membrane
7	DnaK protein	Stress protein	218	4.5/66	51	gi:15924570	Membrane
8	PEP-protein phosphatase	Carbohydrate metabolism	60	4.5/63	20	gi:15926670	Membrane
9	PEP-protein phosphatase	Carbohydrate metabolism	30	4.5/63	18	gi:15926670	Membrane
10	Pyruvate kinase	Carbohydrate metabolism	150	4.1/63	25	gi:15924687	transmembrane
11	Prolyl-tRNA synthetase	Cell division	228	4.9/64	54	gi:15924253	transmembrane
12	Prolyl-tRNA synthetase	Cell division	58	4.9/64	13	gi:15924253	transmembrane
13	30S ribosomal protein	Protein synthesis	130	4.4/43	37	gi:15924466	transmembrane
14	Glutamyl- amidotransferase	Aminio acid metabolism	84	4.9/53	25	gi:21283570	transmembrane
15	Glycyl-tRNA synthetase	Cell division	90	4.9/54	20	gi:15924555	Cytosolic
16,17	Elongation factor Tu	Virulence mechanism	214	4.6/43	60	gi:15923538	Membrane
18	Pyruvate dehydrogenase	Virulence mechanism	180	4.8/41	48	gi:15924083	Membrane
19	Phosphoglycerate kinase	Carbohydrate metabolism	120	5.1/42	42	gi:15923763	transmembrane
20	Coenzyme A ligase	Protein synthesis	140	5.0/43	42	gi:21282234	transmembrane
21	DNA-directed RNA polymerase	Transcription and replication	110	4.5/35	38	gi:15925214	Cytosolic
22	Glyceraldehyde-3-PDH	Carbohydrate metabolism	108	4.8/36	44	gi:15923762	Cytosolic
23	Coenzyme A synthase	Carbohydrate metabolism	30	4.80/43	20	gi:49484747	transmembrane
24	Pyrophosphatase	Carbohydrate metabolism	30	4.5/34	15	gi:15924909	Membrane
25	Phosphotransacetylase	Others	102	4.6/35	55	gi:15923578	Cytosolic
26	Elongation factor Ts	Transcription and replication	190	5.0/32	58	gi:15924247	Cytosolic
27	General stress protein Ctc	Stress protein	50	4.2/24	29	gi:15923491	Cytosolic
28	30S ribosomal protein	Protein synthesis	106	5.3/30	30	gi:15924035	transmembrane
29	Triosephosphate isomerase	Carbohydrate metabolism	58	4.7/27	32	gi:15923764	Cytosolic
31	Superoxide dimutase	Stress protein	80	5.0/23	35	gi:15924543	Cytosolic
32	Cysteine synthase	Carbohydrate metabolism	60	5.2/33	24	gi:15923503	transmembrane
33	Malate:quinone oxidoreductase	Carbohydrate metabolism	220	6.1/56	38	gi:15925597	Membrane
35	50S ribosomal protein	Protein synthesis	174	4.5/13	96	gi:15923530	transmembrane
38	50S ribosomal protein	Protein synthesis	210	9.8/20	77	gi:15925228	Cytosolic
39	50S ribosomal protein	Protein synthesis	78	9.9/20	52	gi:15925225	Cytosolic
40	50S ribosomal protein	Protein synthesis	50	10.8/16	43	gi:15925221	Cytosolic
41	50S ribosomal protein	Protein synthesis	70	9.5/15	42	gi:15923527	Cytosolic
42	50S ribosomal protein	Protein synthesis	40	10.3/11	50	gi:15924637	Cytosolic
36,37,43	50S ribosomal protein	Protein synthesis	10	9.5/24	87	gi:15923528	Cytosolic
44	Endopeptidase	Protein degradation	438	5.4/91	57	gi:15923515	Cytosolic
45	30S ribosomal protein	Protein synthesis	100	10.1/24	44	gi:15925234	Cytosolic
46	Inositol-monophosphate DH	others	178	5.5/53	45	gi:15923380	transmembrane
47	50S ribosomal protein	Protein synthesis	80	10.2/24	36	gi:15925240	Cytosolic
48	Catalase	Stress protein	164	5.2/58	40	gi:21282950	Cytosolic
49	Pyruvate dehydrogenase E1	Virulence mechanism	90	4.8/41	24	gi:15924083	Membrane
50	Threonine dehydrogenase	Aminio acid metabolism	166	6.4/37	35	gi:15924428	Membrane
51	Poly.phosphorlase/adenylase	Others	268	4.7/77	40	gi:15924264	transmembrane
52	Glutamyl-tRNA synthetase	Cell division	116	5.0/56	31	gi:15923518	transmembrane
53	50S ribosomal protein	Protein Synthesis	60	10.3/24	17	gi:15925240	Cytosolic
54	ABC transporter	Cell division	100	5.37/48	32	gi:15923833	transmembrane
55	Adenylosuccinate synthetase	Others	150	5.0/48	41	gi:15923007	Membrane
56	Alkyl hydroperoxide reductase	Stress protein	160	4.6/55	32	gi:15923370	transmembrane
57	Akaline shock protein 23	Stress protein	156	5.0/20	48	gi:15925172	Cytosolic
58	Hypothetical protein SAV1845	Others	90	4.1/13	72	gi:15924835	Cytosolic
59	Acetate kinase	Carbohydrate metabolism	176	5.6/44	61	gi:21283383	transmembrane
60	Alcohol dehydrogenase	Carbohydrate metabolism	140	5.2/36	48	gi:21282297	transmembrane
61	Succinyl-CoA synthetase	Carbohydrate metabolism	46	5.4/32	16	gi:15924236	Not clear
62	Fructose-1,6-bioP- aldolase	Carbohydrate metabolism	190	4.7/33	50	gi:15925596	Cytosolic
63	Acid decarboxylase	Carbohydrate metabolism	240	4.7/33	78	gi:15925596	Cytosolic
64	Fructose-bi-P-aldolase	Carbohydrate metabolism	70	4.9/31	20	gi:15925115	Not clear
65	Phenylalanyl-tRNA synthetase	Aminio acid metabolism	60	4.9/22	37	gi:15924732	Cytosolic
66	Glycerate dehydrogenase	Carbohydrate metabolism	60	5.0/35	30	gi:15925295	Membrane
67	Malate-dehydrogenase	Carbohydrate metabolism	50	4.9/44	17	gi:15924692	Membrane
68	Ornithine-acid transaminase	Aminio acid metabolism	90	5.0/43	40	gi:15923947	Membrane
30,34,69	Alkyl hydroperoxide reductase	Stress protein	30	4.7/21	26	gi:15923371	Cytosolic

Gels similar to those shown in Figure 1 were subjected to image analysis to identify differentially expressed proteins. These spots were then cut from the gels, and proteins were identified via tandem mass spectrometry (MS). Protein identification was performed against NCBInr (nr.gz) databases for *Staphylococcus* genus. Cross correlation (XC) score is a metric associated with the quality of identification, with a score >20 indicating a positive identification; pI/Mr is isoelectric point and molecular weight (theoretical); Cov (%) is the fraction of protein sequence coverage identified via MS; Access. No. is accession number for the protein.

Proteins visualized in the 2-DE profile were also classified based on their functions using PSORT and TMpred. About 30% of the identified proteins were involved in carbohydrate metabolism, 20% in protein synthesis and 12% in stress tolerance. KEGG prediction confirmed the functional categories of three virulence mechanism associated proteins: fibronectin-binding protein (spot 1), collagen-binding surface protein (Cna; spot 4) and trigger factor (spot 5). Fibronectin-binding protein in *S. aureus* is central to the invasion of endothelium. During infection, *S. aureus* forms a bridge by using fibronectin-binding proteins with the host cell receptors [29]. The collagen binding protein (Cna; spot 4) identified in this study is well studied as well for its binding with collagen [32-34]. Unlike fibrinogen- and collagen-binding proteins, the associations of elongation factor Tu (spots 16 and17) and pyruvate dehydrogenase (spot 18) with virulence mechanism could not be predicted by KEGG. On the contrary, association of these proteins with virulence mechanism were reported from other findings [35].

### Effect of fluid shear rate on protein expression in biofilms

**Figure 2:**
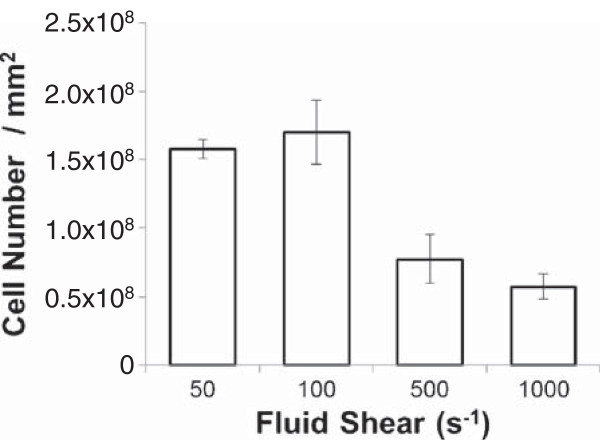
**Number of biofilm cells collected from flow chamber system at each of four different shear rates (Average ± standard deviations).** Four biological replicates were carried out at each shear rate. As described in Methods section, biofilm cells were grown for 24 hrs with continuous supply of sterile tryptic soybroth.

Fluid shear, a hydrodynamic force also generated by blood flow against the vessel wall, is an important physiological phenomenon in the vasculature system [36-38]. Using physiologically relevant fluid shear rate conditions, adequate number of biofilm cells could be harvested to carry out 2-DE for protein separation from both cytoplasmic and membrane cell fractions (Figure 2).

**Figure 3:**
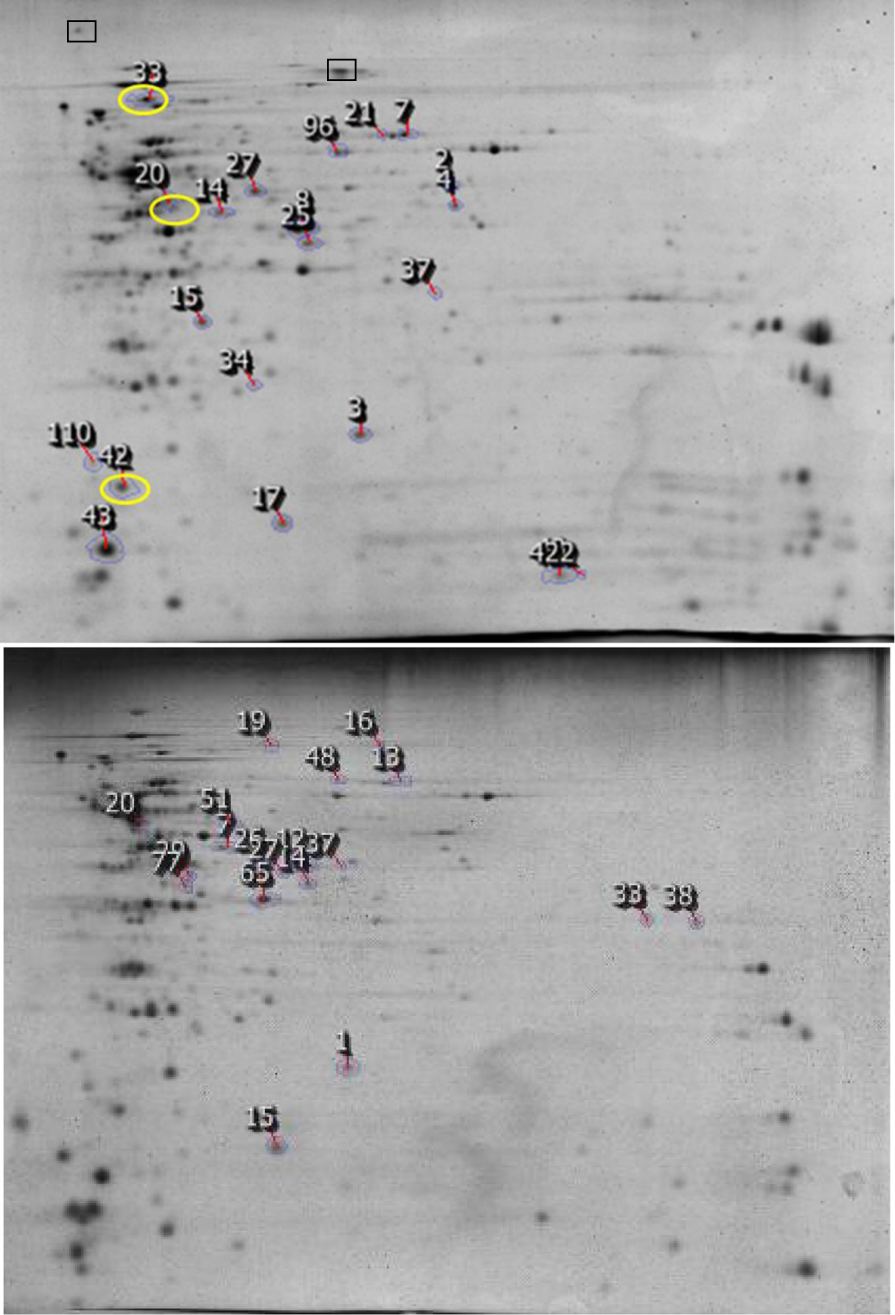
**2-DE gel images of membrane associated fraction (top) and cytosolic fraction (bottom) of *S. aureus *biofilm cells grown at fluid shear rate of 1000 s**^**-1**^**.** Circled spot ID numbers correspond to the proteins with increased level of expression at 1000 s^-1^. Differentially expressed protein were identified and shown in Table 2. Corresponding position of fibronectin-binding protein (spot 1 in Figure 1) and collagen-binding protein (Cna; spot 4 in Figure 1) are shown in rectangular box.

Fluid shear rates range from 40 s^-1^ to 2,000 s^-1^ in physiological conditions [39].To investigate the effect of fluid shear on protein profile in biofilm, the protein expression patterns of biofilm cells grown at 50 s^-1^, 100 s^-1^, 500 s^-1^ and 1000 s^-1^ were compared. Of the 200 protein spots resolved in 2-DE gels from the biofilm cells grown in shake flasks, 16 protein spots showed statistically significant differences in their abundance as shear rate was increased (*p* < 0.05, Student’s *t*-test). These proteins were identified using nano-LC-MS/MS. Their localization and possible functions were predicted by PSORT and TMpred (Figure 3). All the proteins with a differential spot volume in membrane fraction were confirmed as either membrane or transmembrane in their sub-cellular localization. Similarly, eight cytosolic proteins that displayed lower abundance with an increase in the fluid shear rates were identified. Sub-cellular location and function of these proteins in cytoplasm were also confirmed by PSORT and TMpred.

**Figure 4:**
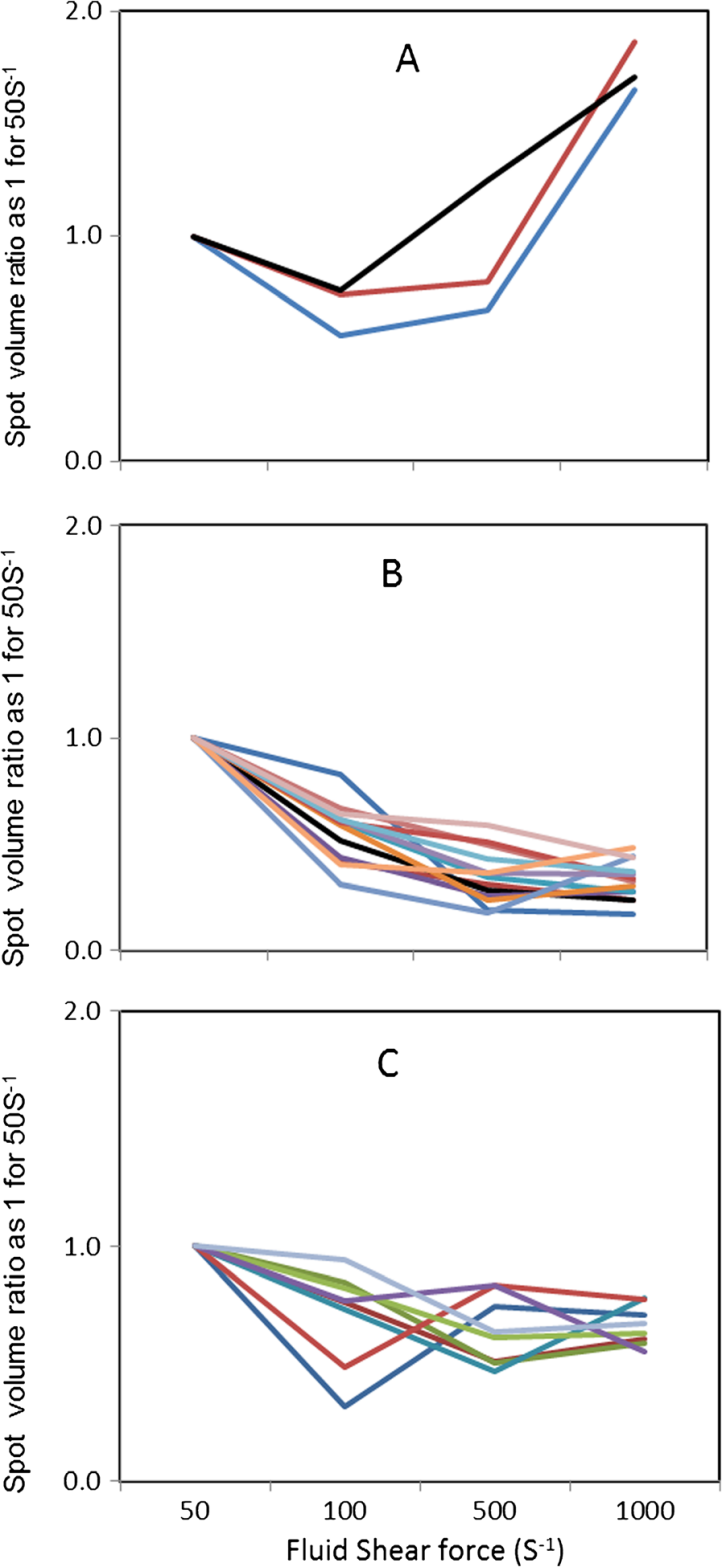
**Protein expression as a function of fluid shear forces.** Number shown in the bracket below corresponds to Spot ID in Figure 3. **A**: membrane fraction (20, 33, 42); **B**: membrane fraction (2, 3, 4, 7, 8, 14, 15, 17, 21, 25, 27, 34, 37); **C**: Cytosolic fraction (1, 7, 12, 14, 19, 20, 26, 33).

As shown in Figure 4, three membrane proteins showed higher abundance in biofilm cells at 1000 s^-1^ when compared to those at 50 s^-1^,100 s^-1^, and 500 s^-1^. These proteins were identified by LC-MS/MS as malate dehydrogenase (spot 20), branched-chain alpha-keto acid dehydrogenase (spot 33) and 50S ribosomal protein (spot 42; Figure 4 and Table 2). Unlike the membrane associated fraction, cytoplasmic proteins that showed higher abundance with an increase in the fluid shears could not be detected.

**Table 2:** Differentially expressed membrane and cytosolic protein fractions were identified with linear ion trap nano-LC-ESI-MS/MS

**Spot ID**	**Fold changed**	**Protein name**	**XC score**	**Cov (%)**	**pI/Mr**	**Acc no**	**Funtional category**
	**Membrane fraction: higher abundance in 1000S-1**						
20	-3.1	Malate dehydrogenase	170	54	4.9/44	gi:15924692	Carbohydrate metabolism
33	-2.5	Brached -chain alpha-keto acid dehydrogenase	260	42	4.8/46	gi:15924085	Carbohydrate metabolism
42	-2.2	50S ribosomal protein	80	58	4.6/17	gi:15923529	Amino acid metabolism
	**Membrane fraction: lower abundance in 1000S-1**						
2	5.8	Glyceraldehyde 3-phosphate dehydrogenase	106	30	5.9/37	gi:15924677	Carbohydrate metabolism
3	4.2	Glyceraldehyde 3-phosphate dehydrogenase	48	21	5.9/37	gi:15924677	Carbohydrate metabolism
4	4.2	Alanine dehydrogenase	258	66	5.5/40	gi:21283381	Carbohydrate metabolism
7	3.6	Malate:quinone oxidoreductase	443	62	6.1/56	gi:15925597	Carbohydrate metabolism
8	3.6	Alcohol dehydrogenase	510	76	5.2/36	gi:21282297	Carbohydrate metabolism
14	3.3	Ornithine--oxo-acid transaminase	340	81	5.1/43	gi:15923947	Amino acid metabolism
15	3.2	Ribosomal subunit interface protein	200	68	5.0/22	gi:15923742	Amino acid metabolism
17	3.1	Nucleoside diphosphate kinase	90	61	4.9/17	gi:15924459	Nucleotide Metabolism
21	2.9	Malate:quinone oxidoreductase	236	44	6.1/56	gi:15925597	Carbohydrate metabolism
25	2.8	Succinyl-CoA synthease	166	49	5.4/32	gi:15924236	Carbohydrate metabolism
27	2.7	Alanine dehydrogenase	148	47	5.0/40	gi:15924429	Carbohydrate metabolism
34	2.5	Superoxide dismutase	118	62	5.2/23	gi:15923123	Stress protein
37	2.4	F0F1 ATP synthase	80	43	5.7/32	gi:15925094	Carbohydrate metabolism
	**Cytosolic fraction: lower abundance in 1000S-1**						
1	0.71	Glyceraldehyde 3-phosphate dehydrogenase	48	21	5.9/37	gi:15924677	Carbohydrate metabolism
7	0.60	Ornithine--oxo-acid transaminase	340	81	5.1/43	gi:15923947	Amino acid metabolism
12	0.59	Alcohol dehydrogenase	516	76	5.2/36	gi:21282297	Carbohydrate metabolism
14	0.78	Succinyl-CoA synthetase	106	30	5.4/32	gi:15924236	Carbohydrate metabolism
19	0.77	Formate acetyltransferase	148	22	5.2/84	gi:15923216	Carbohydrate metabolism
20	0.63	Malate dehydrogenase	170	54	4.9/44	gi:15924692	Carbohydrate metabolism
26	0.55	Alcohol dehydrogenase	510	76	5.2/36	gi:21282297	Carbohydrate metabolism
33	0.67	50S ribosomal protein	80	36	10.2/24	gi:15925240	Amino acid metabolism

Fold change was calculated using proteins in 50 s^-1^ compared to those in 1000 s^-1^. Protein functional categories were annotated from KEGG database.

Proteins that showed decreased expression with increasing fluid shear rates were also observed. Thirteen membrane associated proteins showed lower expression in biofilm cells at 1000 s^-1^ when compared to those at 50 s^-1^,100 s^-1^ and 500 s^-1^ (Figure 4 and Table 2). A closer look at the functional categories of proteins revealed that this decrease in protein expression represented a regulated state of lower metabolic activity. Nine of the 13 identified proteins are associated mainly in glycolysis/TCA cycle, indicating that utility of the glycolysis/TCA pathways were minimized under high shear rates. Glyceraldehyde 3-phosphate dehydrogenase decreased (spot 2 and 3) by 4.2 to 5.8 folds in 1000 s^-1^ when compared to that in 50 s^-1^.

Similar to phosphate dehydrogenase, alanine dehydrogenase (spot 4), another important protein that catalyzes the NAD-dependent reversible reductive amination of pyruvate into alanine, was decreased by 4.2 fold in 1000 s^-1^ when compared to 50 s^-1^ (Table 2 and Figure 4). In addition, a significant decrease in malate:quinone oxidoreductase and succinyl-CoA synthase were observed under high fluid shear rates.

In cytosolic fraction, eight proteins showed lower abundance at 1000 s^-1^ when compared to 50 s^-1^. Like with the membrane protein fraction, glyceraldehyde-3-phosphate dehydrogenase decreased significantly at 1000 s^-1^ when compared to 50 s^-1^. In addition, a lower abundance of alcohol dehydrogenase, succinyl-CoA synthetase, formate acetyltransferase, and malate dehydrogenase was observed under high fluid shear stress.

In the proteome profile reported here, six virulence-associated surface proteins were identified: fibronectin-binding protein (spot 1), collagen-binding surface protein (Cna; spot 4), trigger factor (spot 5), elongation factor Tu (spots 16 and17) and pyruvate dehydrogenase (spot 18). However, changes in expression of these proteins were not detectable under fluid shear stress. On the contrary, most of the glycolysis / TCA cycle associated proteins decreased significantly under higher shear rates. The data thus demonstrate for the first time the relationship between metabolic activity and virulence potential. The glycolysis/TCA metabolic activity of *S. aureus* was low under fluid high shear, while the abundance of surface proteins, especially the fibronectin-binding and collagen binding surface proteins, remained unchanged. Although the ability of these cells in neo-colonization was not specifically tested, the unchanged abundance of these surface binding proteins implies that these cells retain their ability for further infection. Alternatively, there might be an interaction between virulence mechanism associated proteins and glycolysis/TCA associated proteins under high fluid shear that is yet to be determined. As glucose is the main metabolite of the glycolysis/TCA pathways, it is anticipated that carbohydrate metabolism might have potential contribution to the virulence mechanism associated proteins. Such an interaction of carbohydrate structures and their associated proteins with cell wall-anchored proteins was predicted from other findings [25].

### Metabolic pathways of identified proteins

**Figure 5:**
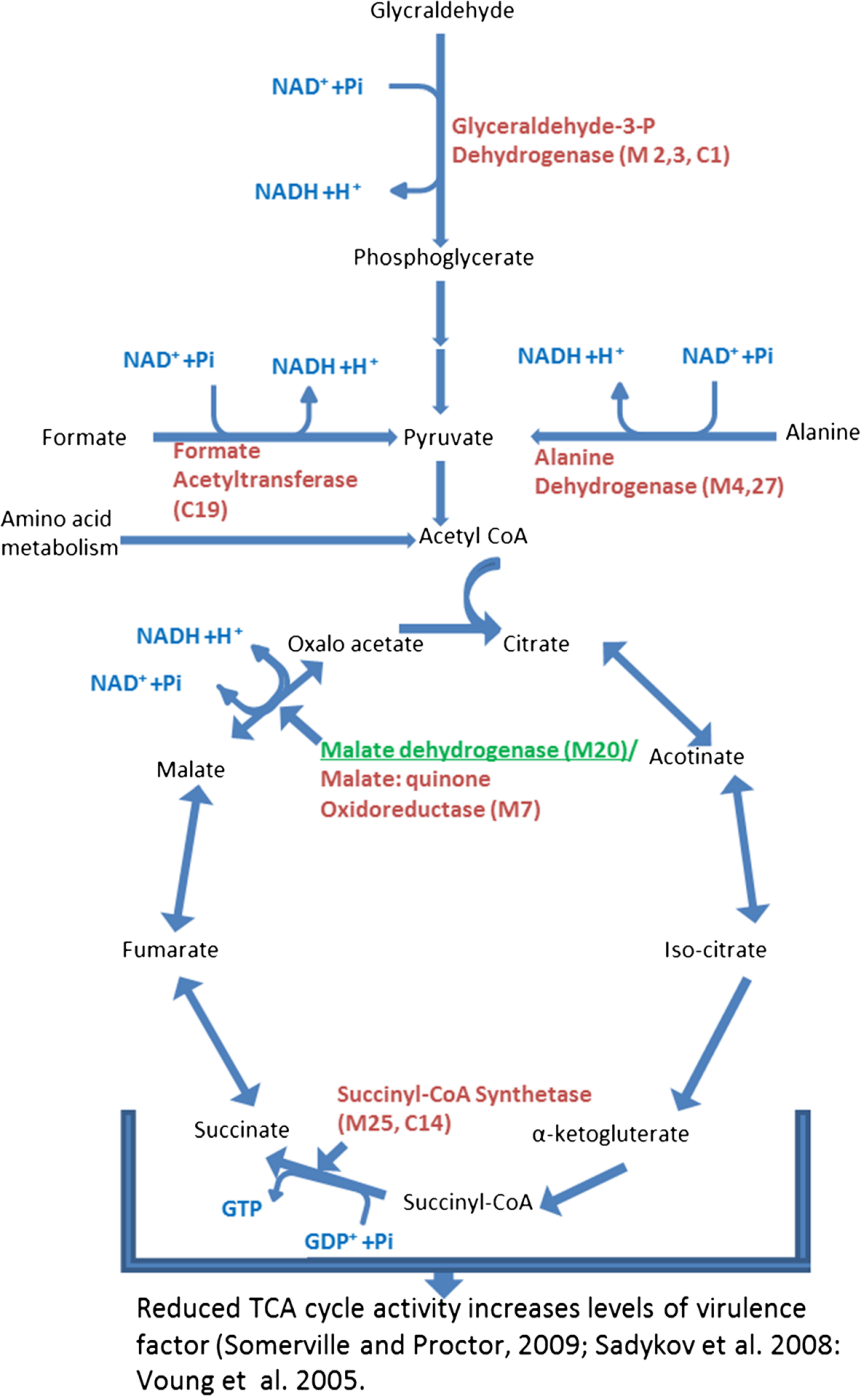
**Metabolic pathways (glycolysis and TCA cycle) of differentially expressed proteins.** Bolded names denote proteins with decreased abundance at 1000 s^-1^ when compared to 50 s^-1^, 100 s^-1^ and 500 s^-1^, while underlined names denote proteins with increased expression. Lower abundance of key enzymes in the TCA cycles increased levels of virulent factors [41,43,45].

Some of the identified proteins were mapped in their metabolic pathways (Figure 5). As observed in Table 2, most of the changed proteins were associated with carbohydrate metabolism especially glycolysis and TCA cycle. Glyceraldehyde 3-phosphate dehydrogenase (GapC; spots M2 and M3, C1) reduced significantly as fluid shear rates were increased. This protein is responsible for the inter-conversion of 1,3-diphosphoglycerate and glyceraldehyde-3-phosphate (GAPDH), a central step in glycolysis which utilizes NAD for phosphorylation. As fluid shear significantly decreased the abundance of this protein, less energy is anticipated to be available for the transfer of inorganic phosphate to high phosphoryl transfer product, indicating that the bacteria exhibits lower metabolic activity under higher fluid shear stress. Although cytosol is considered to be a primary location for this protein, it was recently reported that GAPDH and several other glycolytic enzymes assemble in complexes on the inside of the cell membrane [26]. This protein is also considered as a metabolic switch for rerouting the carbohydrate flux to counteract stress [40].

In addition to a reduction in GAPDH under high fluid shears, significant decreases in alanine dehydrogenase and formate acetyltransferase were also observed. Both of these enzymes provide pyruvate for glycolytic pathways. Reduction in these enzymes also implies lower acetyl-CoA that drives TCA cycles.

There were significant changes in three enzymes of TCA cycle with the increased rate of fluid shear (Figure 5). Malate dehydrogenase that catalyzes the conversion of malate into oxaloacetate (using NAD+) increased significantly. This increase in malate dehydrogenase seems to contradict with the decreased level of expression of other glycolytic and TCA cycle enzymes in this study. However, the reaction mediated by this enzyme is reversible, indicating that the increased level in malate dehydrogenase might have resulted due to the reverse reaction. This is also consistent with the reduction in malate:quinone oxidoreductase (spot 7). In addition, a significant decrease in the expression of succnyl-CoA synthetase was also observed. Taken together, it can be concluded that fluid shear stress have a significant effect on the abundance of some key enzymes in the TCA cycles, implying lower TCA cycle activity.

Implications of reduced TCA cycle activity in regulating or affecting staphylococcal virulence and/or virulence determinant biosynthesis are well studied [41-45]. Higher amount of capsular polysaccharides in relation to TCA cycle inactivation was reported [41]. It was anticipated that TCA cycle inactivation results lower amount of oxaloacetate for conversion to phosphoenoylpyruvate to be used in gluconeogensis pathway [41].

Inhibiting TCA cycle activity dramatically increases polysaccharides intercellular adhesins (PIA) biosynthesis through genetic regulation of *icaADBC*, a group of genes responsible for production of PIA [43,45]. It is anticipated that regulatory proteins responding to TCA cycle-mediated changes in the metabolic status regulate *icaADBC* transcription, indicating that the TCA cycle acts as a signal transduction pathways for regulating virulence factor synthesis and biofilm formation [41]. Lower glycolytic and TCA cycle activities of bacteria under fluid shear stress is also consistent with lower amount of cell that was harvested at 500 and 1000 s^-1^ (Figure 2).

Here, it is demonstrated that under fluid shear conditions, bacteria maintain a dormant mode of growth by reducing glycolytic and TCA cycles activities for their survival while maintaining their capacity for further infection. From clinical perspective, the dormant mode of growth is considered a major role that bacteria plays to escape the effect of antibiotics [46].

## Conclusions

The expression levels of two important surface binding proteins, fibronectin-binding and collagen-binding proteins, were not influenced by varying levels of physiological fluid shear rates. On the other hand, some of the *S. aureus* proteins that are associated with metabolic functions such as carbohydrates, protein synthesis, and stress tolerance significantly changed their level of expression. These results imply that bacteria maintain slow metabolic activity under high fluid shear stress. To our knowledge, this is the first report that catalogs the differential protein expression under various physiologically relevant fluid shear stress conditions. As revealed from this study, most of the altered proteins were directly related to the energy balance of different metabolic pathways. A future study that elucidates post-translational protein modifications (especially phosphorylation) may provide a further understanding of the highly variable biofilm proteomes under fluid shear forces.

## Methods

### Bacterial culture

*S. aureus* Phillips, a biofilm forming bacteria, was used in this study. The strain was isolated from a patient diagnosed with osteomyelitis and has been extensively studied [9,10,33]. *S. aureus* cultures were started by inoculation (10 μL) from glycerol stocks into tryptic soy broth (TSB; 50 μL) supplemented with 0.25% (w/v) glucose and grown at 37°C with constant rotation at 141 rpm in shake flasks. The growth of the bacterial strains was monitored by measuring the absorbance of the broth at 600 nm on a spectrophotometer. The cells were harvested at mid-exponential phase (OD_600_ = 0.3 to 0.35), centrifuged (4000 rpm) for 15 min at 4°C and resuspended in phosphate-buffered saline (DPBS; 138 mM NaCl, 2.7 mM KCl, pH 7.4). Cell concentrations were determined using a Coulter Multisizer (Beckman Coulter).

### Flow chamber system

A 40 cm silicon tube (SILASTIC) was used as the reactor. Briefly, after sterilization, the reactor tube was filled with collagen type I for coating and washed with sterile PBS after an hour. The reactor was then inoculated with 20-fold diluted mid-exponential phase cells for 10 minutes. The unattached cells were then rinsed out of the silicon tubing with sterile PBS buffer. One end of the reactor was then connected to a continuous supply of TSB using a peristaltic pump which was calibrated for different flow rates to provide the desired shear stresses. The wall shear rate ($\dot{\gamma }$) in inverse seconds, assuming Newtonian fluid behavior and constant density and viscosity, was calculated by the formula: $\dot{\gamma }$ = 32Q/πD^3^, where Q is the volumetric flow rate and D is the tube internal diameter. The equation and further explanation of flow rates and shear stress calculations can be found in a recent review [47]. During all experiments, the entire flow system was maintained at 37°C. After 24 hours, the reactor tube was gently removed from the system. Biofilm cells were extruded by squeezing the reactor tube. The tube was washed three times with phosphate-buffered saline to ensure recovery of all biofilm cells from the tube. After brief sonication, the cell concentration was measured using the Coulter Multisizer.

### Protein extraction

Cells were washed with PBS containing 0.1% sodium azide and then with PBS without azide followed by a brief wash with digestion buffer containing 10 mM Tris HCl, 1 mM EDTA, 5 mM MgCl_2_. Approximately 5 × 10^9^ bacterial cells were resuspended in 1 mL of digestion mixture containing 35% raffinose (Sigma), protease inhibitor cocktail (Roche; 1 tablet/mL of digestion buffer), and lysostaphin (Sigma; 5 units/mL). The cells were incubated in this lysis buffer at 37°C for 30 min. Cell debris was removed by centrifugation at 8,000 *g* for 20 minutes. The digest was kept at -20°C overnight and then centrifuged at 8,000 *g* for 20 min; precipitated raffinose was discarded. The protein solution was subjected to ultrafiltration (Millipore) per manufacturer’s instructions. Membrane proteins were extracted in 2-DE solubilization buffer urea (8 M; Sigma), thiourea (2 M; Sigma), ASB 14 (1%; Sigma) and DTT (1%; Sigma), carrier ampholytes (0.08%; GE Healthcare)] with sonication. Protein concentration in the solution was determined using 2D Quant (GE Healthcare), and the resulting solution was stored at -80°C for 2-DE.

### Two dimensional Gel electrophoresis

In preparation for 2-DE, 150 μg proteins were resolubilized by adding 2-DE solubilization buffer. The mixture was vortexed intermittently until the protein was completely solubilized. The resulting solution was then diluted to the desired volume with destreak rehydration solutions (GE Healthcare). Rehydration of IPG strips (pH 3–11, NL 24 cm; GE Healthcare) with the sample was carried out in the Immobiline Dry Strip Re-swelling Tray (GE Healthcare) according to the manufacturer’s instructions. The rehydrated strips were then subjected to IEF using IPGphor (GE Healthcare) operated at 20°C in gradient mode (97 kVhr). After focusing, the strips were stored at -80°C for later use. Prior to the second dimension SDS-PAGE, IPG strips were equilibrated for 15 minutes in equilibration solution (15 mL) containing 50 mM Tris–HCl, pH 8.8, 6 M urea, 30% w/v glycerol, 2% w/v SDS and traces of bromophenol blue with 10 mg/mL (w/v) of DTT. A second equilibration was carried out for 15 minutes by adding iodoacetamide (25 mg/mL) instead of DTT in equilibration solution. Second dimension SDS-PAGE was performed using large format (26.8 × 20.5 cm) gels (12.5% T/2.6% C) according to the manufacturer’s instructions. Electrophoresis was carried out with an initial constant voltage of 10 mA/gel applied for 30 minutes followed by 20 mA/gel for overnight until the bromophenol band exited the gel. The gels were stained with Colloidal Coomassie brilliant blue (BioRad). Briefly, gels were washed two times using distilled water and then placed on Dodeca high throughput gel staining tray (BioRad) followed by destaining using distilled water. All steps were carried out at room temperature. Gels were scanned as 12-bit TIFF images using GS-800 densitometer (BioRad) and analyzed by Nonlinear Dynamics SameSpots (v.3.2). Spot volumes were normalized by the software to a reference gel. At least three gels (biological replicates) for each treatment were used for analyses. Student’s *t*-test was performed, and spots with more than 2-fold were considered as significant.

### Protein identification

For mass spectrometric identification, gel spots were excised, destained, and digested with sequencing grade trypsin (Promega) as described elsewhere [48]. Peptide samples were then analyzed by nano-LC-ESI-MS/MS using LTQ (Finnigan, Thermo, USA). Nano-LC was performed at reversed phase conditions using Ultimate 3000 (Dionex corporation, USA) C18 column with a flow rate of 1–5 μL/min in 70-90% acetonitrile containing 0.1% formic acid. MS and MS/MS data were collected and interrogated against the NCBI non-redundant protein database for *S. aureus* using SEQUEST with a peptide tolerance of 1.4 amu. Searched results were then filtered using three criteria: distinct peptides, XC score vs Charge state (1.50, 2.00, 2.50, 3.00) and peptide probability (0.001). The confirmation of the protein identification was based on the XC score value of more than 50 and Sf score for individual peptide of more than 0.8. It is to be noted that more than one protein were found in each spot, which is not unusual for a gel-based protein separation. Therefore, protein with the highest XC for each spot was selected as positive identification.

### Protein localization and function

The prediction of protein localization sites in cells was determined by PSORT, a computer program which analyzes the input sequence by applying the stored rules for various sequence features of known protein sorting signals. The transmembrane protein domain was predicted by TMpred (http://www.ch.embnet.org/software/TMPRED_form.html). To analyze functional categories of the identified proteins, they were submitted to the KEGG database (http://www.genome.ad.jp/kegg/pathway.html) using BRITE hierarchy. KEGG BRITE is a collection of hierarchical classifications of proteins based on their biological function.

## Competing interests

The authors declare that there are no competing interests.

## Authors’ contributions

NI designed and conducted the experiment and is the corresponding author of this manuscript. JMR and MRM developed the concept. NI and YK analyzed the data and wrote the manuscript. All authors read and approved the final manuscript.
